# Coeliac Disease in Elderly Patients: Value of Coeliac Lymphogram for Diagnosis

**DOI:** 10.3390/nu13092984

**Published:** 2021-08-27

**Authors:** Fernando Fernández-Bañares, Sergio Farrais, Montserrat Planella, Josefa Melero, Natalia López-Palacios, Santiago Vivas, Luis Fernández-Salazar, Ana Pilar Lanzarote, Pablo Ruiz-Ramírez, Marta Aguilar-Criado, Judith Vidal, Aureli Esquerda, Cristina Serrano, Concepción Núñez

**Affiliations:** 1 Department of Gastroenterology, Hospital Universitari Mutua Terrassa, 08221 Terrassa, Spain; pruiz@mutuaterrassa.es; 2 Centro de Investigación Biomédica en Red de Enfermedades Hepáticas y Digestivas (CIBERehd), Instituto de Salud Carlos III, 28029 Madrid, Spain; 3 Department of Gastroenterology, Hospital Fundación Jiménez Díaz, 28040 Madrid, Spain; SFarraisV@quironsalud.es (S.F.); aplanzarote@gmail.com (A.P.L.); 4 Department of Gastroenterology, Hospital Universitari Arnau de Vilanova, 25198 Lleida, Spain; mplanella.lleida.ics@gencat.cat; 5 Department Immunology and Genetics, Hospital Universitario de Badajoz, 06080 Badajoz, Spain; melero.pepa@gmail.com (J.M.); marta.aguilarc@salud-juntaex.es (M.A.-C.); 6 Department of Gastroenterology, Hospital Clínico San Carlos, IdISSC, 28040 Madrid, Spain; natalia.lopa@gmail.com; 7 Department of Gastroenterology, Complejo Asistencial Universitario de León, 24071 León, Spain; svivasa@gmail.com; 8 Department of Gastroenterology, Hospital Clínico Universitario de Valladolid, Gerencia Regional de Salud (SACYL), Facultad de Medicina, Universidad de Valladolid, 47002 Valladolid, Spain; lfernandezsa@saludcastillayleon.es; 9 Department of Flow Cytometry, CATLAB, 08232 Viladecavalls, Spain; jvidal@catlab.cat; 10 Department of Clinical Laboratory, Hospital Universitari Arnau de Vilanova, 25198 Lleida, Spain; aesquerda.lleida.ics@gencat.cat; 11 Department of Clinical Immunology, Hospital Fundación Jiménez Díaz, 28040 Madrid, Spain; CSerranoC@quironsalud.es; 12 Laboratorio de Investigación en Genética de Enfermedades Complejas, Hospital Clínico San Carlos, Instituto de Investigación Sanitaria Hospital Clínico San Carlos (IdISSC), 28040 Madrid, Spain; conchita.npardo@gmail.com

**Keywords:** coeliac, TCRγδ+, CD3−, flow cytometry, elderly

## Abstract

(1) Background: Although a meta-analysis reported that the sensitivity of CD3+ TCRγδ+ cells for coeliac disease diagnosis was >93%, a recent study has suggested that sensitivity decreased to 65% in elderly patients. (2) Aim: To evaluate whether the sensitivity of intraepithelial lymphocyte cytometric patterns for coeliac disease diagnosis changes with advanced age. (3) Methods: We performed a multicentre study including 127 coeliac disease patients ≥ 50 years: 87 with baseline cytometry (45 aged 50–59 years; 23 aged 60–69 years; 19 aged ≥ 70 years), 16 also with a follow-up cytometry (on a gluten-free diet); and 40 with only follow-up cytometry. (4) Results: In Marsh 3 patients, a sensitivity of 94.7%, 88.9% and 86.7% was observed for each age group using a cut-off value of TCRγδ+ >10% (*p* = 0.27); and a sensitivity of 84.2%, 83.4% and 53.3% for a cut-off value >14% (*p* = 0.02; 50–69 vs. ≥70 years), with difference between applying a cut-off of 10% or 14% (*p* = 0.008). The TCRγδ+ count in the ≥70 years group was lower than in the other groups (*p* = 0.014). (5) Conclusion: In coeliac patients ≥ 70 years, the TCRγδ+ count decreases and the cut-off point of >10% is more accurate than >14%.

## 1. Introduction

Coeliac disease (CD) is a common disorder affecting more than 1% of the population in the world [1]. Serologic screening allows the detection of individuals with atypical or subtle symptoms, and even asymptomatic cases [1]. The condition is often assumed to principally affect children and young adults, but it also occurs in elderly people [2,3,4,5,6]. Approximately a quarter of all diagnoses are now made at the age of 60 years or more, and a fifth at 65 years or over [5]. There is some evidence that elderly people with newly detected CD are more often seronegative than younger people [7,8] and that they have greater heterogeneity in clinical presentation, often being misdiagnosed with IBS several years prior to CD diagnosis [6]. Likewise, symptoms of anaemia or weight loss may prompt a malignancy diagnostic work-up prior to considering CD. A low index of suspicion by a physician may lead to diagnostic delay, mainly in seronegative cases [6,8]. However, studies support a rise in the number of CD diagnoses made in advanced aged individuals despite the large aged-based variations in prevalence [3,5], probably due to an increased awareness that this condition may occur in elderly people.

Immunological studies assessing intraepithelial lymphocyte (IEL) subpopulations by flow cytometry in fresh mucosal duodenal samples have described a coeliac intraepithelial lymphogram, defined as an increase in CD3+ T-cell receptor gamma-delta+ (TCRγδ+) IELs plus a concomitant decrease in CD3− IELs, which is highly accurate for CD diagnosis [9,10]. A 2019 meta-analysis showed that the sensitivity of either an increase in TCRγδ+ cells or the presence of the coeliac lymphogram for CD diagnosis was >93% [11]. We have demonstrated the usefulness of this tool for the diagnosis of CD in seronegative villous atrophy [12]. Accordingly, TCRγδ+ IEL count assessed by flow cytometry has been advocated as a new and accurate diagnostic tool for doubtful CD cases, irrespective of the degree of mucosal damage, sex and age [13]. However, in the aforementioned study, the number of CD patients older than 61 was very small, which precluded drawing conclusions regarding elderly people. In fact, a recent study has suggested that the sensitivity of this assay decreased to 65% in a series of patients with a median age of 53 [14]. Using a cut-off of ≥14% for TCRγδ+ cells, these authors showed that CD patients with normal TCRγδ+ IEL percentages were significantly older compared with those with increased TCRγδ+ IEL counts. Specificity, in contrast, was very high regardless of age [11,14].

The recent European Society for the Study of Coeliac Disease (ESsCD) guidelines for CD stated, in the areas of uncertainty and future research, that further studies are needed to validate T cell flow cytometry and make it widely available for clinical use [1]. Thus, the aim of this study was to confirm whether the sensitivity of the flow cytometry IELs pattern for CD diagnosis changes with advanced age and to assess the best diagnostic cut-off for TCRγδ+ cells in elderly people. In addition, we aimed to assess whether the long-term persistence of CD IEL cytometric patterns after a gluten-free diet (GFD) depends on age at onset.

## 2. Materials and Methods

### 2.1. Study Design

This was a retrospective multicentre study searching the CD registries of the seven participating tertiary centres for patients diagnosed with CD who met the following inclusion criteria: (1) age at diagnosis ≥ 50 years, and (2) IELs pattern determined by flow cytometry at baseline (while on a normal gluten-containing diet).

CD diagnosis was based on the presence of positive serology (either IgA anti-tissue transglutaminase -tTG- or IgA anti-endomysium antibodies -EmA-; IgG anti-tTG antibodies were used in case of selective IgA deficiency), Marsh 1 or Marsh 3 histological damage, and a clinical and serological remission after a GFD [1]. Seronegative Marsh 3 patients were diagnosed with CD based on the presence of both a coeliac pattern on IEL flow cytometry and a clinical and histological response to GFD [12,15]. Demographic data, clinical presentation, concomitant diseases, coeliac genetics (HLA-DQ2.5/DQ2.2/DQ8), duodenal histology, TCRγδ+ and CD3− IEL percentage, and clinical and histological response to GFD were recorded for all included patients. All study data were collected and managed using REDCap electronic data capture tools hosted at the Instituto de Investigación Sanitaria Hospital Clínico San Carlos (IdISSC), Madrid, Spain. When flow cytometry and histology data were available after a follow-up biopsy, they were also recorded.

### 2.2. Ethical Issues

The protocol study was approved by the Ethical and Research Committees of the participating centres and the study was conducted in accordance with the Declaration of Helsinki. Retrospective data were coded, and the centres’ corresponding Ethical Committees confirmed that, given the retrospective nature of the non-interventional medical record review, the need to request consent from patients was exempted. Researchers guaranteed strict measures for preserving patient confidentiality.

### 2.3. Flow Cytometry Analysis

For IEL flow cytometry, one single duodenal biopsy from the second portion of the duodenum was obtained and processed immediately as previously described [13]. The results of the flow cytometry were obtained in the four hours following sample collection, which was always before pathology diagnosis. All centres used similar gating strategies to select both CD3− and TCRγδ+ cells, which were measured as CD45 + CD103 + CD3− and CD45 + CD103 + TCRγδ+, respectively, over the total of CD45 + CD103+ cells. We performed comparative studies in parallel on samples from different hospitals and concordance for both CD3− and TCRγδ+ cells was almost 100% in terms of absolute percentages (see Appendix A).

Different cut-offs were used in the different participating centres, ranging from >8.5 to 12%, in most of them >10%, for TCRγδ+ and <10% for CD3− cells, which entails slight variations in sensitivity and specificity between them, i.e., the lower the cut-off the higher the sensitivity, and vice versa. Some centres consider specificity, and others sensitivity, to be more important. The meta-analysis of the published studies using these cut-offs showed a pooled sensitivity and specificity of 95% [11]. Recently, using TCRγδ+ > 10% and CD3− < 10%, we showed 87% of sensitivity and 97% of specificity for seronegative CD [12]. Therefore, we preferred to use the quantitative individual values and interpret them with a single cut-off value that allows for high specificity, thereby maintaining good sensitivity. In the present study, we compare the sensitivity of a cut-off for TCRγδ+ cells of either >10% or >14% (used in the study by Nijeboer et al. [14]) for CD diagnosis. Coeliac lymphogram was then defined as an increase in TCRγδ+ cells according to each cut-off plus a concomitant decrease in CD3− cells <10%. There were four IEL patterns: a normal pattern, an isolated decrease in CD3−, an isolated increase in TCRγδ+ and the coeliac lymphogram (an increase in TCRγδ+ plus a decrease in CD3−) [13]. The last two patterns are associated with CD.

### 2.4. Coeliac Serology

Serum IgA tTG (or IgG tTG in IgA deficient patients) was analysed using homologated commercial quantitative automated ELISAs while the patients were on a gluten-containing diet. Depending on the centre, EliA Celikey IgA (Thermo Fisher, Phadia AB, Uppsala, Sweden), Aeskulisa tTg-A (Aesku.Diagnostics, Wendelsheim, Germany) or Bioplex 2200 Celiac IgA (BioRad, Hercules, CA, USA) were used. Serum EmA was tested by an IF assay at dilution 1:5 in commercial sections of primate distal oesophagus as the antigen substrate (Immco Diagnostics, Buffalo, NY, USA) to confirm a positive result in all samples analysed with the Aeskulisa tTg-A kit. In the remaining centres, EmA was tested in patients with either borderline anti-tTG titres or detectable anti-tTG titres, but below the cut-off suggested by the manufacturer.

### 2.5. Histological Studies

Two endoscopic biopsies from the bulb and four from the second portion of the duodenum were obtained in separate vials for standard histological studies. Duodenal samples were processed using haematoxylin/eosin staining and CD3 immunophenotyping and were evaluated blind to the results of flow cytometry. Marsh 1 was considered as an IEL count of >25 IELs per 100 epithelial nuclei and normal villous architecture [13].

### 2.6. Statistical Analysis

Results are expressed as mean ± SD, median and the interquartile range (IQR) or as percentages. Chi-square statistics were used to compare qualitative variables and the Kruskal–Wallis test was used to compare quantitative variables. McNemar’s test was used to compare the CD diagnosis sensitivity of the two TCRγδ+ cut-offs. Statistical analysis was conducted using the SPSS for Windows statistical package (SPSS Inc., Chicago, IL, USA) and MedCalc statistical software, version 18.2.1 (MedCalc Software bvba, Ostend, Belgium).

## 3. Results

### 3.1. Patients

A total of 127 patients were included (age, 60.7 ± 9.3 years; 79.5% women), 83.3% of whom were HLA-DQ2.5+ and 95.2% had positive coeliac serology. A total of six patients had seronegative CD with villous atrophy.

A total of 87 patients had a baseline flow cytometry assay (16 showing Marsh 1 and 71 Marsh 3) and were divided into three age groups: 45 patients of 50–59 years (mean age, 54.1 ± 2.8 years); 23 of 60–69 years (mean age, 64.6 ± 3.0 years); and 19 ≥70 years (mean age, 75.8 ± 4.7 years). A total of 16 of these patients also had a follow-up cytometry (on a GFD). A total of 40 additional patients only had a follow-up flow cytometry assay on a GFD (with four showing Marsh 1 and thirty-six Marsh 3 at diagnosis). Median follow-up time for these 56 patients with a follow-up biopsy was of 2 years (IQR, 1.25 to 6). Of patients, 26.5% had persistent villous atrophy despite being in clinical and serological remission after a GFD.

### 3.2. Clinical Phenotype

We compared the clinical phenotype at presentation between the different age groups in 123 patients (four patients were excluded from this analysis due to lack of data). We categorised the clinical presentation of CD as either malabsorption or non-malabsorption features, regardless of “classical” or “non-classical” nomenclature as previously suggested [3] (Table 1). The proportion of patients presenting with at least one malabsorption feature was 70%. *Diarrhoea* and iron deficiency *anaemia* were the most commonly presented malabsorption features. There was a trend to a higher prevalence of IBS-type and dyspepsia symptoms in the youngest age group (*p* = 0.054, 50–59 vs. ≥70 years). There were no significant differences in the remaining features between age groups. In addition, there were no differences in the clinical presentation that guide CD suspicion and diagnosis (Supplementary Figure S1).

### 3.3. Flow Cytometry Pattern at Diagnosis

**Figure 1:**
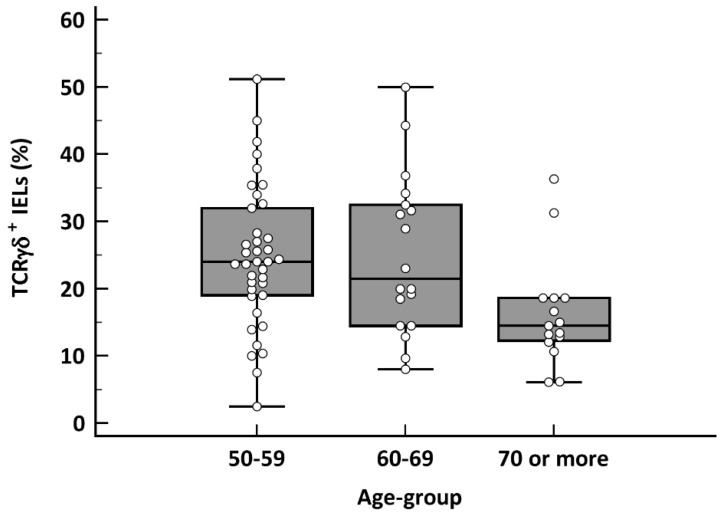
Box-plot chart of TCRγδ+ cells in CD patients with villous atrophy (*n* = 71), depending on the different age groups (*p* = 0.009; Kruskal–Wallis test).

**Figure 2:**
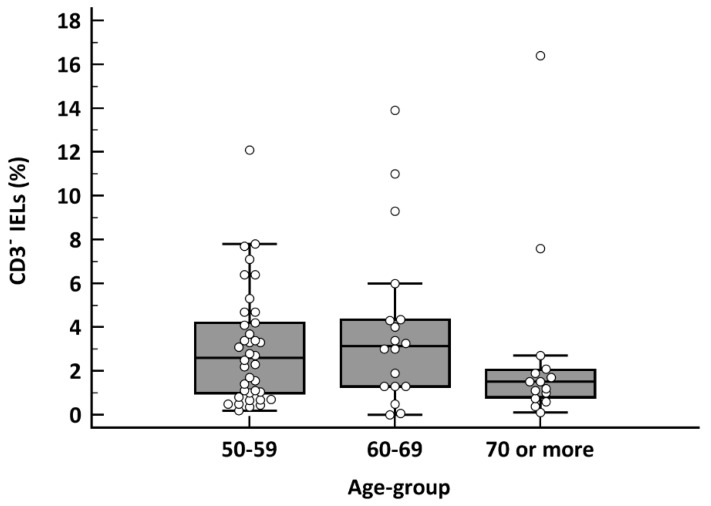
Box-plot chart of CD3− cells in CD patients with villous atrophy (*n* = 71), depending on the different age groups (*p* = 0.25; Kruskal–Wallis test).

In patients with villous atrophy (*n* = 71), a cut-off of TCRγδ+ >10% in the baseline flow cytometry had a sensitivity for CD diagnosis of 94.7%, 88.9% and 86.7% for each age group (*p* = 0.27) (Table 2). In the case of TCRγδ+ >14%, sensitivity was 84.2%, 83.4% and 53.3% (50–69 vs. ≥70 years: *p* = 0.02) (Table 2). These results were similar when considering the coeliac lymphogram. Statistically significant differences were found when comparing the sensitivities observed with the two cut-offs (*p* = 0.008). The TCRγδ+ IEL count in the ≥70 years group was significantly lower than in the other age groups (*p* = 0.009) (Figure 1). There were no differences in the CD3− cell count (*p* = 0.25) (Figure 2).

In patients with Marsh 1 type damage (*n* = 16), a cut-off of TCRγδ+ >10% showed a sensitivity of 85.7%, 100% and 75% for each age group (*p* = 0.16), whereas for a >14% cut-off sensitivity was 71.4%, 80% and 75%, respectively (*p* = 0.44). Overall, a TCRγδ+ >10% cut-off had a sensitivity of 86.9%, and a TCRγδ+ >14% cut-off had a sensitivity of 75.5% (*p* = 0.13) (Table 3).

### 3.4. Flow Cytometry Pattern at Follow-Up

**Figure 3:**
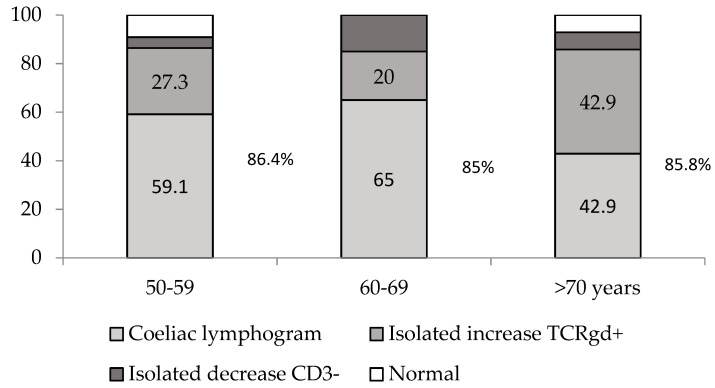
Flow cytometry patterns after a median of two years on a gluten-free diet, depending on the different age groups (*n* = 56).

As mentioned, 56 patients were included in this analysis. In total, 86% of them maintained an increase in TCRγδ+ >10% after a median of two years on a GFD, with no differences between the age groups (86.4%, 85% and 85.8%) (Figure 3). There were no significant differences between patients with villous atrophy persistence and those with mucosal recovery.

## 4. Discussion

The results of the present study confirm that the increase in TCRγδ+ cells count at diagnosis is lower in CD patients aged 70 years and over than in younger patients. This was observed clearly in patients with a Marsh 3 lesion at diagnosis; for Marsh 1 patients, the small number of patients in each age group precludes the possibility of drawing robust conclusions. Therefore, the diagnostic cut-off for TCRγδ+ >10% is more appropriate for this age group than >14%, which was the cut-off used in the Nijeboer et al. study [14]. In that study, a >14% cut-off was associated with a sensitivity of 65%, which is lower than that previously described [11]. As mentioned above, the cut-off used for TCRγδ+ cells count may vary between different laboratories, depending on whether specificity or sensitivity is considered to be more important. In general, this produces slight variations but does not change the non-coeliac/coeliac classification of the IEL pattern in young people. However, as is shown in the present study, that is not the case in patients aged 70 years and over. Notwithstanding, CD diagnosis sensitivity when using a >10% cut-off remains very high, being 87% in the ≥70 years group and 90% in the overall group. We have not evaluated the test specificity in the present study since it was very high regardless of age in previous studies [11,13,14]. We have also shown the high diagnosis accuracy of this test for CD in seronegative villous atrophy of different aetiologies [12].

Over the past years, it has been shown that CD may start at any age and that it may present with subtle and variable clinical expressions. The pattern of clinical presentation in our study was not different in patients ≥70 years to that of adults aged 50 to 69 years. The development of gastrointestinal symptoms such as *diarrhoea*, abdominal pain and distension was the most common presentation that led to the diagnosis. Often, these symptoms were compatible with a functional bowel disease such as IBS or functional dyspepsia. Likewise, iron-deficient *anaemia* was frequently the guide manifestation for CD diagnosis. In studies comparing the clinical presentation in elderly patients and young adults, elderly patients presented with significantly fewer symptoms in some studies [3], though others have described a similar clinical presentation [16,17]. In our study, the frequency of a GI clinical presentation in the form of *diarrhoea*, IBS-like symptoms, dyspepsia and/or bloating was similar to the frequency reported in the aforementioned studies [16,17].

Although the analysis of coeliac lymphogram is not needed for CD diagnosis in seropositive patients, it may have high diagnostic value in doubtful cases that involve differentiating between CD and non-CD atrophy, those in which the mucosal lesion is equivocal or when serum tTG levels are negative or with low titres [13]. In addition, it has been shown that TCRγδ+ IELs remain elevated in CD patients despite a GFD [11]. The persistence of increased TCRγδ+ cell values after a long-term GFD opens the possibility of using this biomarker to confirm CD in patients who have started on a GFD, and for whom serology and histology yield misleading results. In addition, it may prove useful for distinguishing between CD and non-coeliac gluten sensitivity in patients who are symptom-free after a GFD and reluctant to undergo a gluten challenge. The results of the present study show that the long-term persistence of CD IEL cytometric patterns after a GFD is independent of age at onset. In fact, 86% of patients maintained an increase in TCRγδ+ IELs >10% after a median of two years of follow-up, which is in line with previous studies [11]. This is the only existing test capable of reliably diagnosing CD in the presence of gluten withdrawal, non-atrophic enteropathy and negative serology.

Remarkably, although their presence has been described for a number of years, the role of intestinal TCRγδ+ subsets in the pathogenesis of CD is not completely understood. It has been suggested that these cells play roles in regulation and repair within the small intestine [18,19,20,21]. They also have a number of properties that distinguish them from conventional T cells. These include the ability to secrete epithelial growth factors and to produce innate cytokines and chemokines that recruit inflammatory cells [18,20]. Furthermore, they may play an essential role in promoting epithelial restitution following mucosal injury. In this sense, it has been suggested that intestinal TCRγδ+ IELs play a multifaceted role in maintaining mucosal homeostasis following injury, and that there is a dynamic and reciprocal crosstalk between the intestinal microbiota and TCRγδ+ T cells [21], thus acting as the front-line defence system against some antigens [22]. It has also been suggested that distinct subsets of TCRγδ+ IELs can accumulate during the various stages of CD [23]. In the gut of active CD patients, effector TCRγδ+ IELs may predominate, in conjunction with an increased production of IL-15 and IL-21. In contrast, when gluten is withdrawn from the diet and IL-15 and IL-21 levels diminish, TCRγδ+ IELs may become regulatory via the production of the immunosuppressive cytokine TGF-β1 and may therefore contribute to recovery from gluten-driven epithelial damage [24,25]. This could explain why TCRγδ+ IELs remain elevated in the coeliac gut long after the removal of gluten from the diet and the resolution of intestinal damage [19,25].

## 5. Conclusions

In conclusion, the TCRγδ+ IELs count decreases in CD patients ≥70 years, with a TCRγδ+ cut-off >10% being more accurate than >14% for CD diagnosis (sensitivity, 87% vs. 53%). TCRγδ+ IELs remain elevated in the long term despite a GFD. Therefore, the TCRγδ+ IELs count may be of diagnostic use in those cases where diagnosis is not straightforward, regardless of age.

## Figures and Tables

**Table 1:** Association between malabsorption features at presentation and age at CD diagnosis.

Malabsorption Feature	Age 50–59 Years (*n* = 62)	Age 60–69 Years (*n* = 37)	Age ≥70 Years (*n* = 24)	*p*-Value
At least one feature	42 (67.7%)	27 (73%)	17 (70.8%)	0.85
Diarrhoea	16 (25.8%)	14 (37.8%)	7 (29.2%)	0.46
Iron-deficiency anaemia	18 (29%)	13 (35.1%)	10 (41.7%)	0.51
B12 deficiency	3 (4.8%)	2 (5.4%)	1 (4.2%)	1
Folate deficiency	1 (1.6%)	1 (2.7%)	1 (4.2%)	0.76
**Non-Malabsorption Feature**				
IBS-dyspepsia-bloating	32 (51.6%)	13 (35.1%)	8 (33.3%)	0.17
Osteopenia/osteoporosis	9 (14.5%)	4 (10.8%)	1 (4.2%)	0.48
Dermatitis herpetiformis	4 (6.5%)	4 (10.8%)	0	0.26
Hypertransaminasemia	3 (4.8%)	3 (8.1%)	2 (8.3%)	0.70
Other	6 (9.7%)	4 (10.8%)	6 (25%)	0.17

*p*-values were obtained with the Chi-square test.

**Table 2:** CD diagnosis sensitivity of the two coeliac cytometric patterns in patients with villous atrophy in function of the age group.

Flow Cytometry CD Pattern **	Age 50–59 Years (*n* = 38)	Age 60–69 Years (*n* = 18)	Age ≥70 Years (*n* = 15)	Overall Cohort (*n* = 71)
TCRγδ+ >10%				
Increase in TCRγδ+	94.7%	88.9%	86.7%	90.1%
Coeliac lymphogram *	92.1%	77.8%	86.7%	85.5%
TCRγδ+ >14%				
Increase in TCRγδ+	84.2%	83.4%	53.3%	73.6%
Coeliac lymphogram *	84.2%	77.8%	53.3%	71.7%

* Coeliac lymphogram was defined as an increase in *TCRγδ+* cells according to each cut-off, plus a concomitant decrease in CD3− cells <10%; ** Comparison using the McNemar’s test between the two *TCRγδ+* cut-offs (>10% vs. >14%): *p* = 0.008; comparison using Chi-square test between 50–69 vs. ≥70 years groups for the *TCRγδ+* >14% cut-off: *p* = 0.02.

**Table 3:** CD diagnosis sensitivity of the two coeliac cytometric patterns in patients with Marsh 1 in function of the age group.

Flow Cytometry CD Pattern	Age 50–59 Years (*n* = 7)	Age 60–69 Years (*n* = 5)	Age ≥70 Years (*n* = 4)	Overall Cohort (*n* = 16)
TCRγδ+ >10%				
Increase in TCRγδ+	85.7%	100%	75%	86.9%
Coeliac lymphogram *	85.7%	80%	25%	63.5%
TCRγδ+ >14%				
Increase in TCRγδ+	71.4%	80%	75%	75.5%
Coeliac lymphogram *	71.4%	60%	25%	52.1%

* Coeliac lymphogram was defined as an increase in TCRγδ+ cells according to each cut-off plus a concomitant decrease in CD3− cells <10%.

## Data Availability

The datasets generated and/or analysed during the current study are available from the corresponding author on reasonable request.

## Supplementary Materials

The following are available online at https://www.mdpi.com/article/10.3390/nu13092984/s1, Supplementary Figure S1: Clinical presentations that guide CD suspicion and diagnosis. IDA: iron-deficiency *anaemia*.

## Appendix A. Concordance in Intraepithelial Lymphocyte Analysis between Different Cytometres and Centres

In all the participating centres, development and fine-tuning of the methodology to analyse intraepithelial lymphocytes (IELs) by flow cytometry was performed following the indications of the pioneer lab of this methodology in Spain (Dr Garbiñe Roy, Immunology Department of the Hospital Ramón y Cajal, Madrid, Spain; reference centre). In all cases, several samples were analysed in parallel to confirm proper performance.

Here we describe the comparison of the data obtained in parallel using two different flow cytometers and by one of the participating centres and the reference centre (raw data described in Table A1). A good grade of concordance between cytometers and centres was obtained, especially for TCRγδ+ and CD3− IELs, which are the two parameters with direct implication in CD diagnosis (Table A2, Table A3 and Table A4).

**Table A1:** Intraepithelial lymphocyte subpopulation data obtained using two different flow cytometers by one participating centre (CATLAB) and by the reference centre (Hospital Ramón y Cajal). Percentages are shown (over the total of CD45+ CD103+ cells).

Subject	BD FACSCalibur CATLAB		BD FACSCanto CATLAB		BD FACSCanto Hospital Ramón y Cajal	
	TCRγδ+	CD3−	TCRγδ+	CD3−	TCRγδ+	CD3−
1	0.94	15.88	1.4	15.3	1	16.8
2	5.72	2.52	4.3	4.1	5.5	4.3
3	35.57	5.03	32.1	6.2	40	6.3
4	35.77	6.2	33.1	6.1	36.8	3.4
5	4.05	4.75	4.3	5.2	6.1	5.5
6	17.34	9.64	14.6	10	24.6	8.7
7	12.59	0.98	12.6	1.08	16.9	0.7
8	1.17	40.44	1.2	40.4	1.2	49.5
9	5.27	17.7	5.2	17.5	8.2	26.6
10	5.86	23.03	5.8	24.6	6.8	31.4
11	6.54	11.72	7	11.2	9.3	13.3
12	35.67	4.49	35.5	6.7	38.8	5.4
13	1.92	33.45	1.3	32.5	7.5	31.4
14	16.94	7.4	16.5	8.1	27.5	0.7
15	16.33	2.45	16.3	2.2	28.7	1.5

**Table A2:** Comparison of the different lymphocyte subpopulation data obtained using BD FACSCalibur and BD FACSCanto in the same centre (CATLAB) (*n* = 15). Variable X shows data obtained with the BD FACSCanto cytometer and variable Y shows data obtained with the BD FACSCalibur cytometer.

	CD3−		TCRγδ+	
	Variable X	Variable Y	Variable X	Variable Y
Passing and Bablok regression				
Lowest value	1.08	0.98	1.2	0.94
Highest value	40.4	40.44	35.5	35.77
Arithmetic mean	12.74	12.37	12.74	13.445
Median	8.1	7.4	7	6.54
SD	11.52	11.82	11.98	12.75
SE of the mean	2.9751	3.0523	3.0943	3.2937
Regression Equation	y = −0.617424 + 1.032197x		y = −0.145981 + 1.035514x	
Systematic differences				
Intercept A	−0.6174		−0.146	
95% CI	−1.5254 to −0.07676		−0.6255 to 0.08486	
Proportional differences				
Slope B	1.0322		1.0355	
95% CI	0.9785 to 1.1019		0.9971 to 1.1182	
Random differences				
RSD	0.6455		0.8313	
±1.96 RSD Interval	−1.2652 to 1.2652		−1.6293 to 1.6293	
Linear model validity				
Cusum test for linearity	*p* = 0.92		*p* = 0.99	
Spearman rank correlation coefficient				
Correlation coefficient	0.975		0.969	
Significance level	*p* < 0.0001		*p* < 0.0001	
95% CI	0.924 to 0.992		0.906 to 0.990	

SD = standard deviation; SE = standard error; RSD = residual standard deviation; CI = confidence interval.

**Table A3:** Comparison of the different lymphocyte subpopulation data obtained in two different centres with different flow cytometers (*n* = 15). Variable X shows data obtained in the reference centre (Hospital Ramón y Cajal) with the BD FACSCanto cytometer and variable Y shows data obtained in CATLAB with the BD FACSCalibur cytometer.

	CD3−		TCRγδ+	
	Variable X	Variable Y	Variable X	Variable Y
Passing and Bablok regression				
Lowest value	0.7	0.98	1	0.94
Highest value	49.5	40.44	40	35.77
Arithmetic mean	13.7	12.37	17.26	13.44
Median	6.3	7.4	9.3	6.54
SD	14.59	11.82	14.11	12.75
SE of the mean	3.768	3.0523	3.6436	3.2937
Regression Equation	y = 1.271610 + 0.785593x		y = −0.805921 + 0.796053x	
Systematic differences				
Intercept A	1.2716		−0.8059	
95% CI	−0.9053 to 3.4190		−2.6434 to 0.9249	
Proportional differences				
Slope B	0.7856		0.7961	
95% CI	0.6241 to 0.9991		0.6038 to 0.9875	
Random differences				
RSD	2.5237		2.8749	
±1.96 RSD Interval	−4.9465 to 4.9465		−5.6348 to 5.6348	
Linear model validity				
Cusum test for linearity	*p* = 0.69		*p* = 0.49	
Spearman rank correlation coefficient				
Correlation coefficient	0.877		0.925	
Significance level	*p* < 0.0001		*p* < 0.0001	
95% CI	0.661 to 0.958		0.784 to 0.975	

SD = standard deviation; SE = standard error; RSD = residual standard deviation; CI = confidence interval.

**Table A4:** Comparison of the different lymphocyte subpopulation data obtained in two different centres with the BD FACSCanto cytometer (*n* = 15). Variable X shows data obtained in the reference centre (Hospital Ramón y Cajal) and variable Y shows data obtained in CATLAB.

	CD3−		TCRγδ+	
	Variable X	Variable Y	Variable X	Variable Y
Passing and Bablok regression				
Lowest value	0.7	1.08	1	1,2
Highest value	49.5	40.4	40	35.5
Arithmetic mean	13.7	12.74	17.26	12.74
Median	6.3	8.1	9.3	7
SD	14.5935	11.5223	14.1115	11.9843
SE of the mean	3.768	2.9751	3.6436	3.0943
Regression Equation	y = 1.212500 + 0.791667x		y = −0.107611 + 0.764259x	
Systematic differences				
Intercept A	1.2125		−0.1076	
95% CI	−0.04449 to 3.0357		−1.7135 to 0.8299	
Proportional differences				
Slope B	0.7917		0.7643	
95% CI	0.6786 to 0.9912		0.5701 to 0.9369	
Random differences				
RSD	2.3631		2.7141	
±1.96 RSD Interval	−4.6318 to 4.6318		−5.3196 to 5.3196	
Linear model validity				
Cusum test for linearity	*p* = 0.92		*p* = 0.49	
Spearman rank correlation coefficient				
Correlation coefficient	0.887		0.935	
Significance level	*p* < 0.0001		*p* < 0.0001	
95% CI	0.688 to 0.962		0.811 to 0.978	

SD = standard deviation; SE = standard error; RSD = residual standard deviation; CI = confidence interval.
