# Supplementary material for: Coeliac Disease in Elderly Patients: Value of Coeliac Lymphogram for Diagnosis
Source: Nutrients. 2021 Aug 27;13(9):2984. doi: 10.3390/nu13092984 (PMC8467369; doi:10.3390/nu13092984)
Supplement: Supplementary file 1 [file nutrients-13-02984-s001.zip › nutrients-1313505-supplementary.pdf]

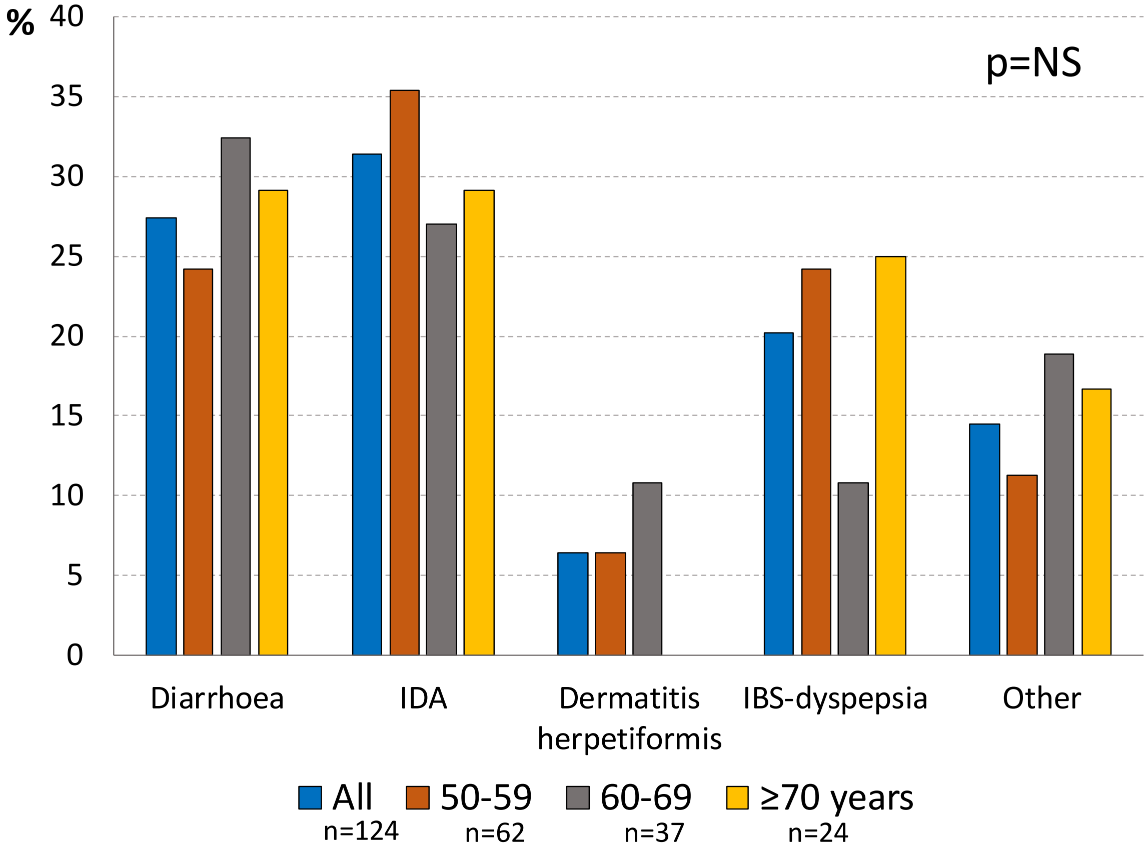

Figure S1. Clinical presentations that guide CD suspicion and diagnosis. IDA: iron-deficiency *anaemia*.
